# Supplementary material for: Three-dimensional beating pattern of the ciliary tip in the live ciliate Tetrahymena
Source: J Cell Sci. 2025 Sep 24;138(20):jcs264027. doi: 10.1242/jcs.264027 (PMC12517402; doi:10.1242/jcs.264027)
Supplement: Supplementary information [file joces-138-264027-s1.pdf]

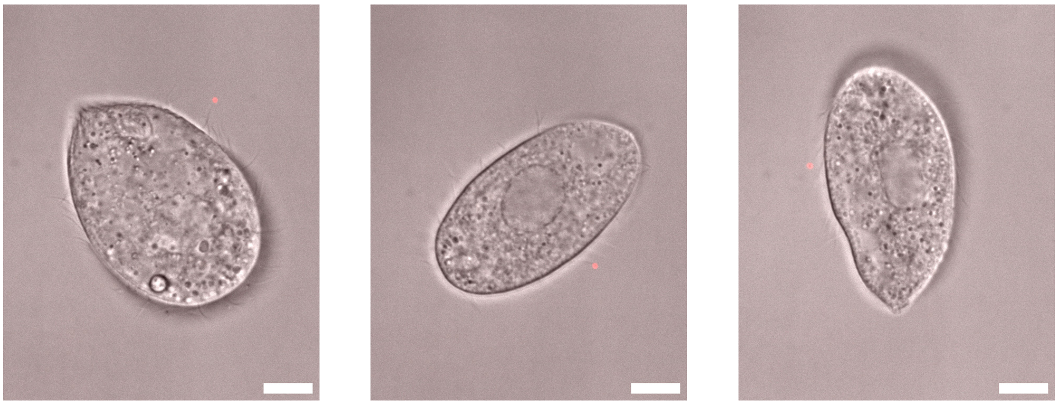

Fig. S1. Fluorescent beads predominantly bind at or near the distal ends of cilia Representative confocal images showing fluorescent neutravidin-coated beads (0.2-µm in diameter) localization on biotinylated cilia of *Tetrahymena*. Red signals indicate fluorescent beads; images are merged with corresponding bright-field images. Cells were fixed with 2% paraformaldehyde (final concentration) after bead attachment and imaged using confocal microscopy (an inverted microscope (IX83, Evident) equipped with spinning disk confocal unit (CSU-X1, Yokogawa Electric)). In all 20 observed cilia (excluding those at the oral apparatus), beads were found to be bound at or near the distal ends of cilia. Bar, 10 µm.

Journal of Cell Science • Supplementary information

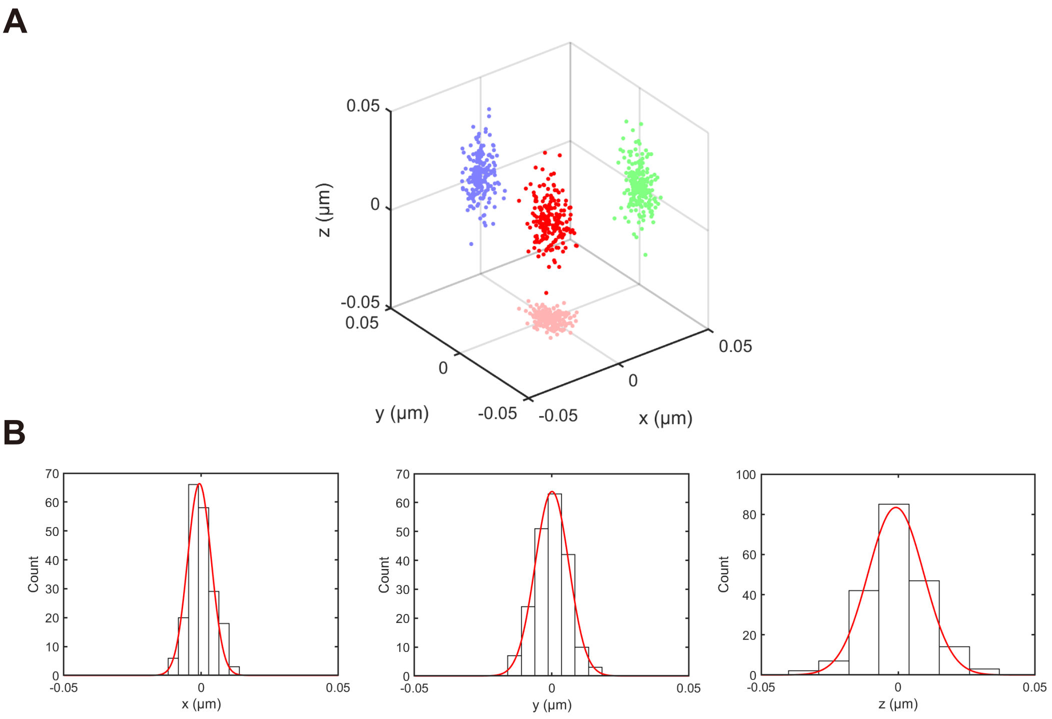

Fig. S2. Tracking uncertainty of 3D measurement using the *tPOT* microscope

A 3D plots show the positions of a 0.2 μm (diameter) microbead fixed in the agarose gel in the observation chamber using a 60×objective (UPLSAPO60XW, 60×, NA 1.20, Olympus, Tokyo, Japan) during 2.2-s recording at 11.21 ms per frame. 3D (red), *x*–*y* (pink), *x*–*z* (blue), and *y*–*z* (green) plots are shown. **B** Histograms of *x*, *y*, and *z* positions of the 0.2 μm microbead shown in (**A**). The standard deviations were derived from a Gaussian fitting (SD; $x = 0.004$ μm, $y = 0.006$ μm, $z = 0.010$ μm).

Journal of Cell Science • Supplementary information

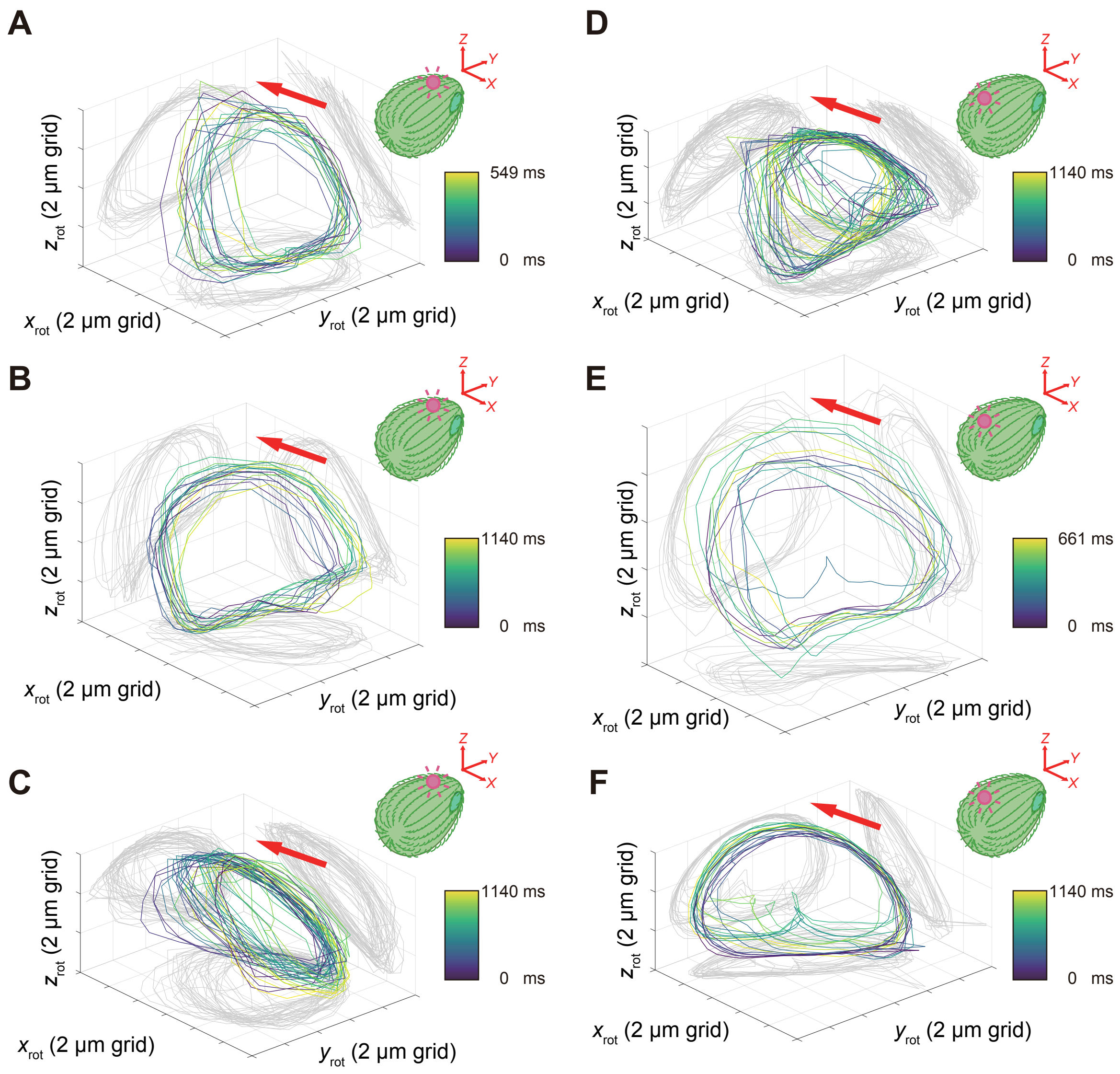

Fig. S3. Additional examples of rotated 3D trajectories of individual ciliary tip movements in live *Tetrahymena* cells

A-F Examples of the trajectories of the individual ciliary tip movement of each cell are shown. The rotated 3D trajectories of the microbeads bound to individual cilium reveal the semi-circled CCW rotational motion viewed from the above. Colour indicates the observation time (see the colour bar). The red arrows indicate the direction of rotation. A few trajectories of the microbead bound to a cilium displayed circular CCW rotational motion (**C**).

Journal of Cell Science • Supplementary information

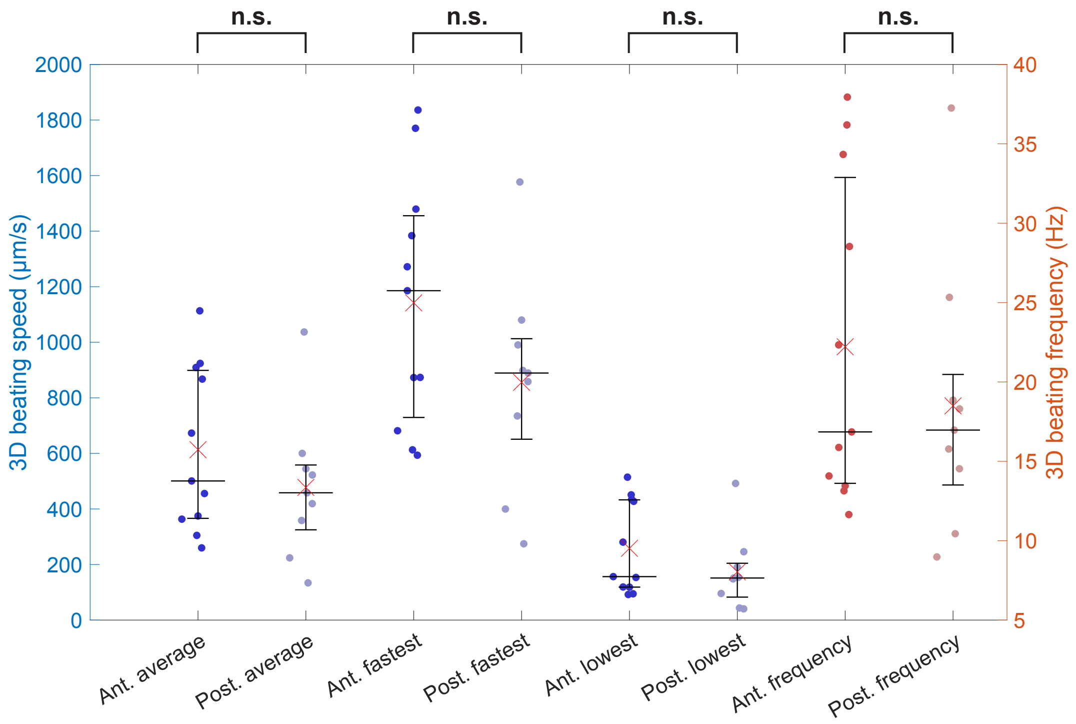

Fig. S4. Comparison of beating parameters between anterior and posterior cilia Swarm plots showing a comparison of 3D ciliary beating parameters between anterior and posterior cilia. Blue plots represent the average, fastest, and lowest speeds of ciliary 3D motion; red plots represent the 3D beating frequency. Each parameter is shown for both anterior (Ant., n = 11) and posterior (Post., n = 9) cilia. Each dot corresponds to an individual cilium. Horizontal lines indicate the first quartile, median, and third quartile. Red crosses indicate the mean values (Ant. average speed: 613 µm/s; Post. average speed: 477 µm/s; Ant. fastest speed: 1142 µm/s; Post. fastest speed: 856 µm/s; Ant. slowest speed: 258 µm/s; Post. slowest speed: 174 µm/s; Ant. frequency: 22 Hz; Post. frequency: 18 Hz). No statistically significant differences were observed between anterior and posterior cilia for any parameter, as determined by the Wilcoxon rank-sum test (average speed: p = 0.447; fastest speed: p = 0.288; slowest speed: p = 0.362; frequency: p = 0.649).

Journal of Cell Science • Supplementary information

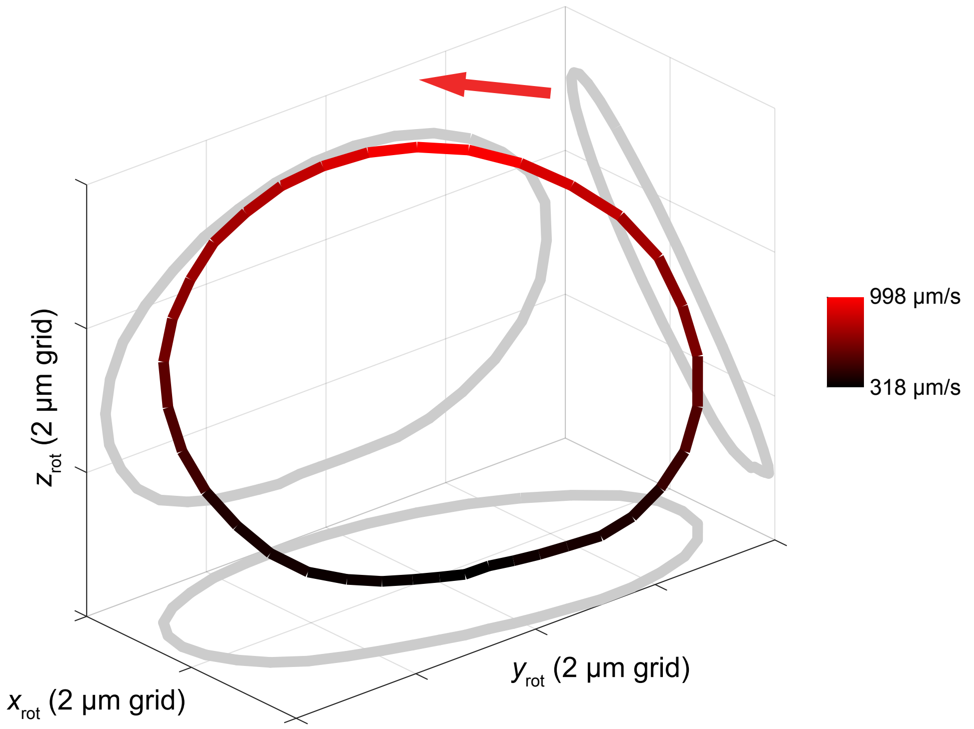

Fig. S5. Averaged 3D trajectory of ciliary tip motion based on 20 individual traces The figure shows the averaged 3D trajectory of ciliary tip motion calculated from 20 individual traces. Trajectory colour represents the average inter-section speed, as indicated by the accompanying colour bar. The red arrow indicates the direction of rotation. All the 3D trajectories of the microbeads bound to each cilium were fitted to a single plane and divided into 36 sections with polar coordinates whose origin was the centroid with declination every 10° and the position vectors and inter-section speeds were calculated (see **Fig. 3A**). To align the trajectories, we identified the three consecutive sections with the highest combined speed in each trace and rotated and translated each trajectory such that the average displacement vector of these three sections aligned with that of the population mean ($n = 20$). The final averaged trajectory was obtained by calculating the arithmetic mean of the position vectors and inter-section speeds across all traces for each of the 36 sections.

Journal of Cell Science • Supplementary information

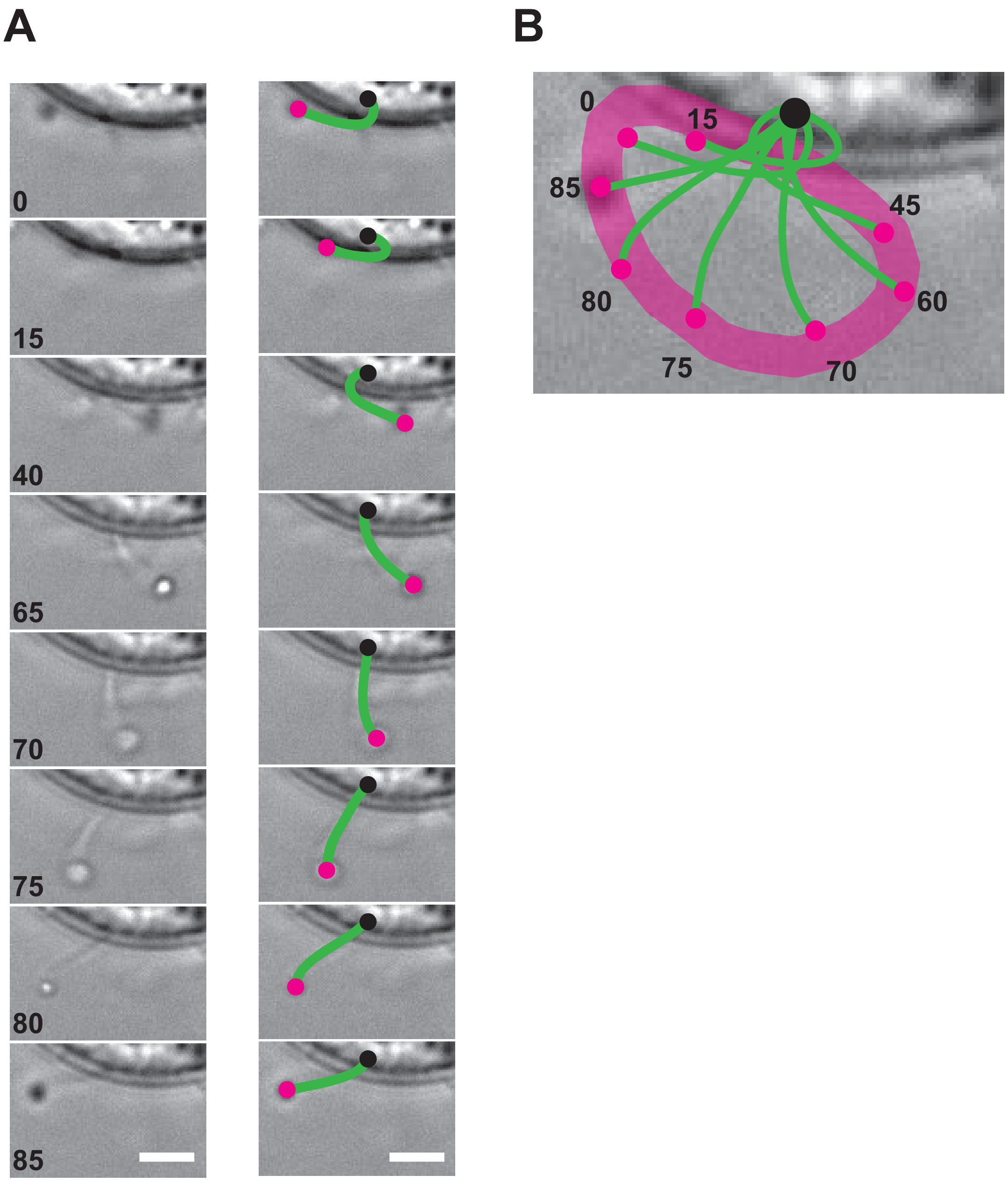

Fig. S6. The shape of one beat cycle of the cilium inferred from the 2D side-view images

A Left; Successive bright-field images showing an example of an individual cilium and a microbead (0.5-µm in diameter, F8812, Thermo Fisher Scientific) bound to the cilium in the live *Tetrahymena* cell captured using a micropipette. Images were recorded by sCMOS camera (Zyla 4.2, Andor Technology, Belfast, UK) via Solis software (Andor) with 1 ms exposure every 5 ms for a typical imaging cycle. Time in milliseconds. Bar, 2 µm. Right: The green line, pink dot and black dot in each frame trace the same cilium, microbead and the base, respectively. Note that cilia during the recovery stroke are located closed to the cell surface and are invisible in most of the recovery stroke. Thus, the shape of cilium and the position of the microbead in some frames were speculated. **B** Superimposed images of **A**.

Table S1.

Available for download at
https://journals.biologists.com/jcs/article-lookup/doi/10.1242/jcs.264027#supplementary-data

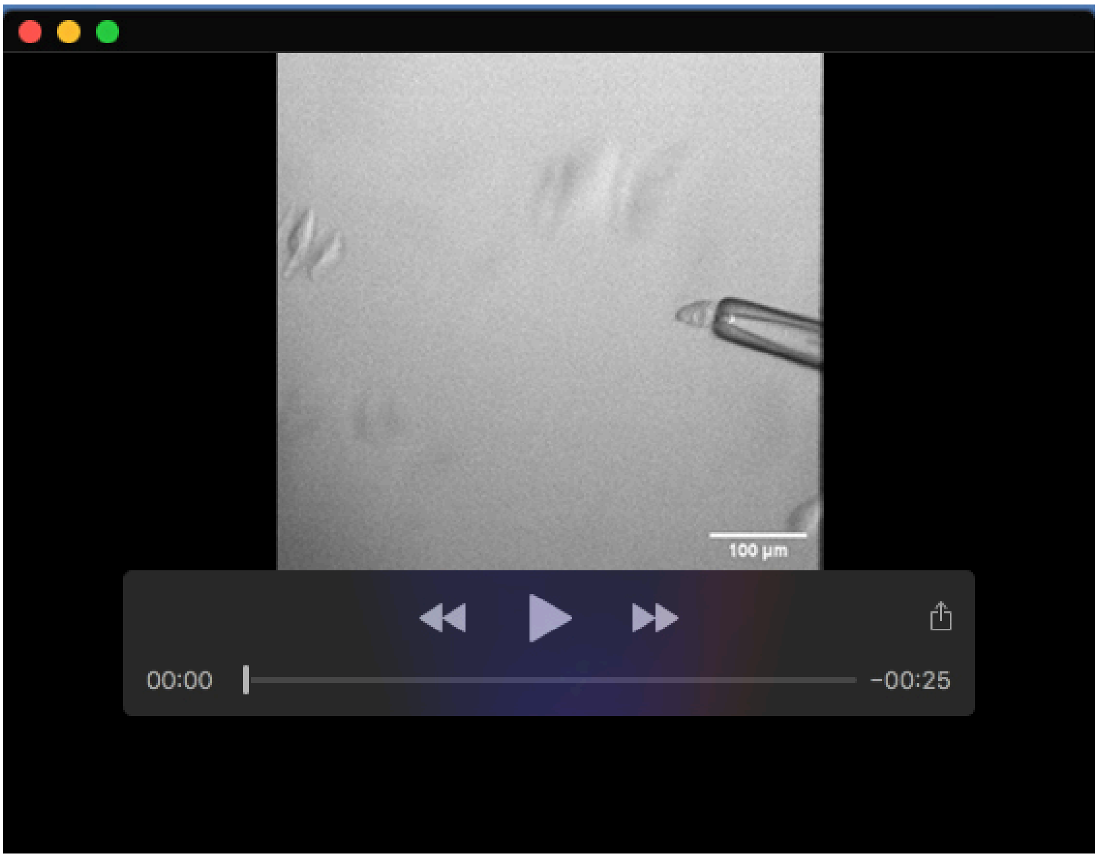

Movie 1. Swimming of *T. thermophila* after being released from the micropipette aspiration. After being released from the micropipette aspiration, the cell swims in a normal helical pattern. This movie shows the swimming cell for ~6.6 seconds (11 ms intervals, normal speed). Scale bar: 100 µm. The image is 565 µm high and 538 µm wide.

Journal of Cell Science • Supplementary information

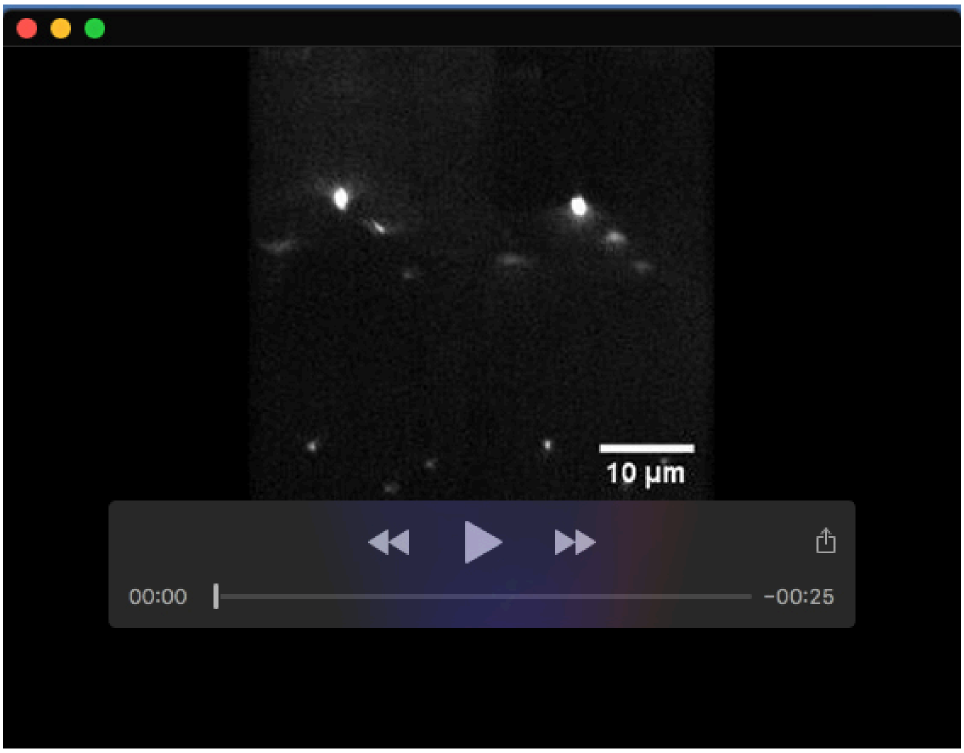

Movie 2. A fluorescent microbead motion bound near the tip of the cilium in the live *T. thermophila* cell trapped by a micropipette. The movement of a fluorescent microbead bound near the ciliary tip was imaged. The movie shows the field of views split by a prism using the *tPOT* system, for ~0.27 seconds (2.28 ms intervals, ×1/20 speed). Scale bar: 10 µm.

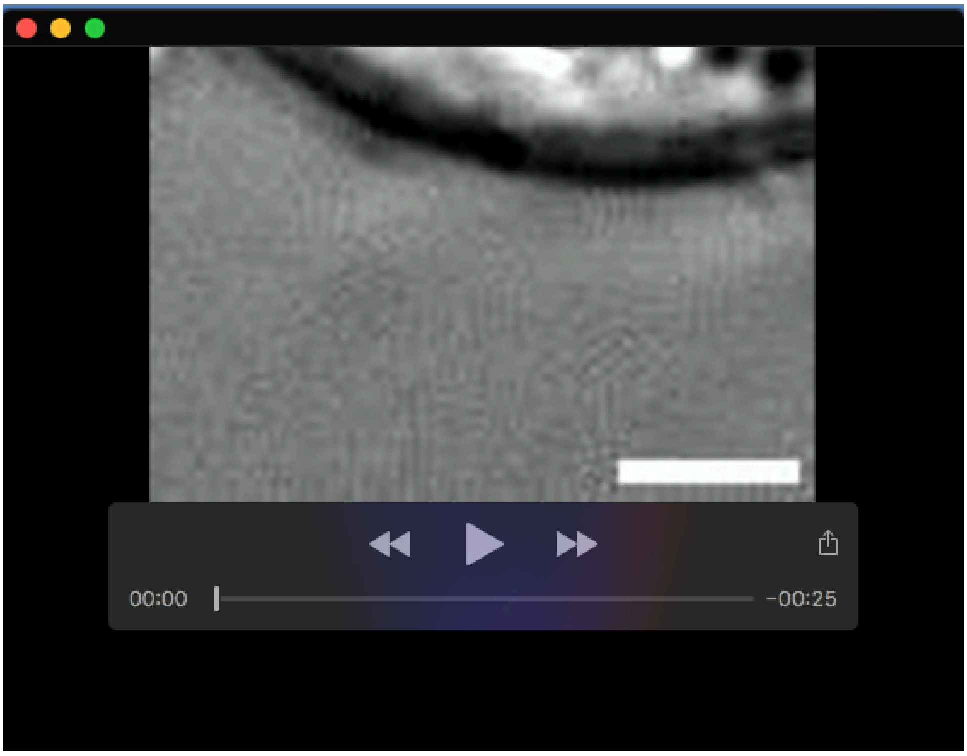

Movie 3. 2D bright-field view of the movement of a microbead bound to the cilium and the shape of the cilium in the live *T. thermophila* cell trapped by a micropipette. One beating of the cilium and the microbead bound near the ciliary tip was imaged. The movie shows 2D blight-field view recorded by sCMOS camera for 105 ms (5 ms intervals, ×1/50 speed). Scale bar: 3 µm.

Journal of Cell Science • Supplementary information
